# Paving the Road for More Ethical and Equitable Policies and Practices in Telerehabilitation in Psychology and Neuropsychology: Protocol for a Rapid Review

**DOI:** 10.2196/66639

**Published:** 2025-04-22

**Authors:** Dorothée Morand-Grondin, Jeanne Berthod, Jennifer Sigouin, Simon Beaulieu-Bonneau, Dahlia Kairy

**Affiliations:** 1 Department of Psychology University of Montreal Montreal, QC Canada; 2 Centre de recherche interdisciplinaire en réadaptation du Montréal métropolitain Montreal, QC Canada; 3 École de psychologie Université Laval Quebec, QC Canada; 4 Centre interdisciplinaire de recherche en réadaptation et intégration sociale, CIUSSS de la Capitale-Nationale Quebec, QC Canada; 5 Centre intégré universitaire de santé et de services sociaux du Centre-Sud-de-l’Ile-de-Montréal (CCSMTL) Montreal, QC Canada; 6 School of Rehabilitation University of Montreal Montreal, QC Canada; 7 Institut universitaire sur la réadaptation en déficience physique de Montréal Montreal, QC Canada

**Keywords:** telerehabilitation, psychology, neuropsychology, equity, ethics, virtual rehabilitation, database, rapid review, Canada, telemedicine

## Abstract

**Background:**

Virtual rehabilitation, or telerehabilitation (TR), has exponentially evolved in the last few years, gaining particular momentum since the COVID-19 pandemic. In response to a new reality of strict restrictions of physical contact necessitating the shift from in-person health services to tele-health visits, TR has seen widespread adoption. In this context, ensuring ethical and equitable TR services is crucial for establishing sustainable TR models for psychology and neuropsychology into health care systems. This requires complete and consistent guidance for clinicians and patients involved.

**Objective:**

The objective of this study is to synthesize existing evidence to provide timely insights on potential ethical and equitable benefits and pitfalls associated with the use of TR in a psychological and neuropsychological framework.

**Methods:**

A rapid review of TR practices will be conducted specifically within the context of neuropsychology and psychology rehabilitation. We will include review articles published between 2010 and 2020 as well as original articles published between 2020 and 2023, all addressing TR issues with a main focus on neuropsychological and/or psychological rehabilitation activities. This research protocol describes the methodology, including search strategy, screening process, data extraction, and analysis methods.

**Results:**

Guided by an experienced librarian, the search strategy was designed and performed in 3 relevant databases. Articles were screened in accordance with the inclusion and exclusion criteria, and data were collected by 2 independent reviewers. Data extraction is underway, and we expect to complete the rapid review in January 2025.

**Conclusions:**

This study is part of a broader cross-Canadian initiative aimed at informing policy development and clinical practices in TR. By evaluating the ethical and equitable considerations specific to psychology and neuropsychology, this review aims to contribute to help shape future TR practices to ensure access to high-quality, accessible TR services supporting diverse patient needs in psychology and neuropsychology.

**International Registered Report Identifier (IRRID):**

DERR1-10.2196/66639

## Introduction

In previous years, the use of telerehabilitation (TR), also known as virtual rehabilitation, has rapidly increased [1-3]. TR is the branch of telehealth whose purpose is to remotely assess, monitor, or provide rehabilitation that best meets the patient’s needs [4]. In the context of the COVID-19 pandemic, the public health system has been compelled to adjust its services in response to the emerging health crisis, which included making necessary changes in care delivery methods [4,5]. To comply with strict distancing regulations and protect vulnerable populations such as older adults and individuals with disabilities and chronic illnesses, TR has been suggested as a promising and viable alternative to traditional in-person health services while ensuring the continued delivery of essential rehabilitation services [6].

TR in neuropsychology and psychology can facilitate the assessment and treatment of psychological symptoms and disorders (eg, anxiety and depression), cognitive functions and impairments (eg, memory and attention), and interventions for psychological and cognitive problems (eg, psychotherapy and cognitive rehabilitation). TR approaches have been shown to be as effective as in-person care and has additionally been suggested as a means to enhance accessibility to health care services [3,7,8]. Indeed, systematic reviews have shown that virtual rehabilitation can be as effective as traditional services for patients with different conditions across their lifespan [9-16]. Furthermore, TR can improve access to care for rural populations, those with mobility challenges and individuals facing demanding work or caregiving responsibilities [7].

Despite its benefits, TR is a care model that is still in development, and as such, inconsistent and incomplete guidance can potentially affect the quality of care provided, especially for vulnerable populations such as individuals from lower socioeconomic backgrounds, those with low digital literacy, and rural populations. Ethical considerations in TR encompass fundamental principles such as integrity, accountability, independence and impartiality, respect for dignity, worth, equality, diversity, and privacy, and professional conduct [17]. In neuropsychology and psychology, these concerns manifest through challenges such as privacy threats due to the presence of family members during interventions, risks in security of sensitive data, or changes in the clinician-patient relationship [18]. Equity is another key concern in telehealth, defined as the absence of unfair, avoidable, or remediable differences between groups [19]. TR risks unintentionally exacerbating social and cultural disparities by requiring patients to have access to private electronic device supporting video consultation, stable high-speed internet, and a private space to conduct the intervention—resources that may be less accessible to underserved populations such as individuals from lower socioeconomic populations and those living in rural areas [20].

Neuropsychology and psychology clinicians, decision makers, and managers must navigate complex considerations associated with the implementation of TR in the COVID-19 and post–COVID-19 environment [21], while individuals with disabilities need support and resources to adapt to this new rehabilitation services model. Although existing research has explored the implementation of telehealth in neuropsychology and psychology, highlighting its feasibility, potential benefits and challenges, significant gaps remain in addressing the ethical and equity-related challenges specific to neuropsychological and psychological interventions within physical telerehabilitation. To harness the potential benefits of TR, it is essential to develop and apply robust guidance and tools addressing concerns related to inadequate and inequitable practices. This includes the need to address significant ethical issues for both clinicians and patients, such as proactively identifying and addressing potential inequities adversely affecting certain populations [22]. This review aims to fill this gap by focusing on the virtual delivery of care in the specific context of neuropsychology and psychology interventions in physical rehabilitation. This focus will provide more in-depth insights into these issues and more appropriately adapted solutions for this field of rehabilitation. By identifying existing barriers and proposing appropriate solutions, we aim to contribute to the development of equitable, evidence-based TR practices in these fields.

The proposed rapid review is one of a series of rapid reviews that are part of a larger cross-Canada study aimed at guiding policy-making and clinical practice to provide ethically sound and equitable virtual rehabilitation care. In order to better inform recommendations, a series of rapid reviews, conducted by field of TR, including in neuropsychology and psychology, are being conducted. Findings from the individual reviews will be combined to inform subsequent steps of the pan-Canadian study (surveys, interviews, and focus groups).

## Methods

### Study Design

A rapid review approach was chosen as the first step of this study. While systematic reviews offer the most comprehensive and rigorous evidence synthesis, they require significant time and resources and can quickly become outdated, particularly in a fast-evolving field such as TR [23]. A rapid review is more suitable for efficiently synthesizing and summarizing evidence in a timely manner [24], providing actionable results for an emerging topic. Given the dynamic nature of TR and the urgency of addressing its ethical and equity considerations, a rapid review ensures that findings remain current and readily available for informed decision-making. This study will provide up-to-date and contextualized scientific and ethical guidance to ensure that TR continues to be integrated into clinical practices and contribute to improve health equity and access to care in Canada.

### Search Strategy

This rapid review is conducted in two main phases: (1) a review of existing reviews on TR in the fields of neuropsychology and psychology and (2) a review of recent original articles in the field. Guided by an experienced health science librarian, an web-based search strategy was designed based on the PICOTSS (Population, Intervention, Comparison, Outcome, Timeframe, Study Design, and Setting) framework. Studies will be included in this review if they meet the following general inclusion criteria: (1) review articles published between 2010 and 2020 or original studies published between 2020 and 2023, (2) articles written in French or English, and (3) articles addressing TR and related terms with their main focus on neuropsychological and/or psychological evaluation and intervention rehabilitation activities conducted with patients with physical disabilities. Only peer-reviewed literature will be included, while gray literature and preprints will be excluded, given their extensive amount deemed unsuitable for the purpose and timeline of this rapid review. The search strategy will be designed for articles related to virtual care using specific keywords related to TR (eg, “telehealth,” “e-health,” “digital health,” “m-health,” and other synonymous terms), neuropsychology, and psychology. Three databases were selected (MEDLINE, CINAHL, and Embase) for their broad scope in medical literature, allowing a comprehensive overview of relevant TR reviews and studies. Reference lists of the included articles were not screened for additional studies, given the time-sensitive nature of this rapid review. The search strategy was designed to comprehensively capture relevant peer-reviewed literature, reducing the necessity of supplementary screening through reference lists. The full search strategy, including the exact syntax and search terms for all databases, is provided in Multimedia Appendix 1.

### Eligibility Criteria

This rapid review follows the PICOTSS framework to ensure a structured study selection based on the research questions identified by the research team (see Table 1). To be included in the rapid review, studies needed to involve human individuals of diverse age groups receiving TR interventions in any setting with neuropsychological and/or psychological services aiming to supplement or replace in-person care. This includes both synchronous and asynchronous approaches, such as videoconferencing, phone calls, recorded videos, links to exercises, or educational materials. Studies needed to report outcomes related to ethical and equity components, efficacy of the intervention, engagement, acceptance, feasibility, and satisfaction, among others. Review articles (including systematic reviews, rapid reviews, narrative reviews, etc) were included from 2010 to 2020, while original study articles (within any design) were included if they were published between 2020 and 2023. These criteria were chosen to balance comprehensiveness and relevance. TR began to gain traction around 2010, with notable technological advancements and increased integration into health care systems shaping its evolution. Considering the scale of virtual rehabilitation literature, exclusively reviews published between 2010 and 2020 will be included to provide a broad and synthesized overview of key developments in the field during this period. This choice ensures that critical findings from earlier studies are incorporated while prioritizing synthesis of evidence and a manageable scope. The inclusion of original articles between 2020 and 2023, including those having been conducted during the COVID-19 pandemic, allow capturing of the most up-to-date advancements in technology, emerging ethical and equity concerns in response to rapid digital transformation, and changes in clinical practice. As information and communication technologies have evolved rapidly and become more readily available in the last few years [25], recent studies are essential to understanding current practices and challenges in TR implementation.

**Table 1:** Criteria for inclusion and exclusion of studies.

PICOTSS^a^ criteria	Inclusion	Exclusion
Population	Human patients of all ages who receive telerehabilitation services with a neuropsychology or psychology intervention.	Animal studies.
Intervention	Telerehabilitation with a neuropsychology or psychology intervention intended to supplement or replace in-person care, including both synchronous and asynchronous interventions.	Studies with no direct interaction with a psychology or neuropsychology health care professional (eg, self-guided interventions without follow-up).
Comparison	Not applicable.	Not applicable.
Outcomes	Any outcome related to ethical and equity components, efficacy, engagement, acceptance, feasibility, and patient satisfaction, among others.	Outcomes not relevant to the review questions.
Timeframe	Studies conducted from January 2010 to March 2023.	Studies conducted before January 2010 and after March 2023.
Setting	All telerehabilitation settings (eg, outpatient, inpatient, home, nursing home, and hospital).	No exclusion.

^a^PICOTSS: Population, Intervention, Comparison, Outcome, Timeframe, Study Design, and Setting.

### Data Collection

Data collection and extraction will take place using the screening and extraction software Covidence (Veritas Health Innovation). After identifying reviews and articles using the keywords, the data collection will follow 3 main steps. First, the reviews and articles will be screened based on their title and abstract. Second, the reviews and articles included in the first step will be examined by the reviewers based on the full text. Third, the data will be extracted from the included reviews and articles in data extraction tables and analyzed with team members for emerging and overarching themes. At each step, an independent verification of the data was performed by a second author on a random sample of 10% of the articles, following established recommendations for rapid reviews [26]. In the rare cases where consensus was not initially reached on inclusion or exclusion, discrepancies were resolved through discussion among all authors until full agreement was reached. Studies included in this review were critically appraised following the methodological guidance from the Cochrane Rapid Reviews—Interim Guidance from the Cochrane Rapid Reviews Method Group [26,27]. Moreover, the Equity-Based Framework for Implementation Research [28], the Quadripartite Ethical Tool [29], and the Consolidated Framework for Implementation Research [30] were used to guide the data collection and analysis. These guidelines provide structured recommendations to maintain rigor and reliability for analyzing barriers and facilitators to health care implementation. In fact, these frameworks provide a comprehensive approach to assess how ethical and equity issues manifest in TR implementation, ensuring that findings were contextualized within broader health care considerations. In accordance with Cochrane’s rapid review guidelines, risk of bias assessment was limited to primary outcomes to optimize the feasibility of the rapid review process. Particular attention was paid to risk of biases in the methods and outcomes that could impact the ethical and equity aspects being examined in this study. By prioritizing risk of bias assessments for these outcomes, this approach ensures a focused evaluation of the most critical evidence while maintaining efficiency in the review process. Risk of bias was assessed by one reviewer and discussed with a second team member. Where discrepancies occurred, they were further discussed with the other team members. For example, the studies’ inclusion and exclusion criteria for articles (for review articles) and study participants (for original articles) were examined for their risk of introducing bias. Lastly, studies were reviewed for potential missing data on key variables. Methodological gaps were noted and considered as part of the risk of bias assessment and reported in the synthesis.

Data will be extracted using a customized data extraction spreadsheet developed by one of the authors based on the study frameworks and adapted to the needs of psychological and neuropsychological fields by two of the authors. Characteristics of interest include (1) the field of therapy, (2) the clinical features (eg, patient population, technology used, outcome measures and tools used, and frequency of intervention), (3) study objectives and findings, (4) limitations (eg, ethical limitations), (5) the quality assessment and risk of bias, and (6) equity considerations.

## Results

This review adhered to PRISMA (Preferred Reporting Items for Systematic Reviews and Meta-Analyses) guidelines (see PRISMA checklist in Multimedia Appendix 2). The search strategy was designed and performed in the 3 identified databases. Initial electronic database searches have retrieved 566 reviews and 2751 original articles meeting the search criteria. Articles have been exported to Covidence and were screened in accordance with the inclusion and exclusion criteria by 2 independent reviewers. Ultimately, 17 reviews and 82 original articles meeting the eligibility criteria were included for data extraction. Preliminary results from data extraction identified key concern dimensions with a focus on ethical and equitable aspects of TR. Ethical concerns centered on privacy, confidentiality, burden of care, and patient-clinician relationship quality and accessibility issues. Equity-related concerns focused on access to care, digital literacy, and socioeconomic disparities issues.

## Discussion

### Anticipated Findings

This paper describes the procedure of the first phase of our study: a rapid review that aims to synthesize evidence on the ethical and equity-related challenges associated with TR in neuropsychology and psychology in the context of physical rehabilitation. Through identification, analysis, and extraction of relevant data, this study will contribute to a more comprehensive understanding of current TR practices and challenges. The findings will serve as a foundation for informing subsequent steps in order to provide guidance to ensure ethically sound and equitable virtual rehabilitation practices. Particularly given the current rapid expansion of TR, it is essential to assess both its benefits and limitations in the context of neuropsychology and psychology. Research has demonstrated the feasibility and effectiveness of web-based neuropsychological and psychological interventions; yet, concerns persist regarding ethical and equity considerations. This study addresses an important need in the field of rehabilitation by contributing to filling a gap in the literature by identifying current challenges and emerging needs based on real-world implementation. Our review identified key ethical and equity-related barriers and facilitators associated with TR. Ethical concerns centered on privacy, confidentiality, the clinician-patient relationship, and potential burden of care. These concerns align with those reported in existing literature, which emphasizes that web-based neuropsychology and psychology interventions must address heightened risks related to data security, professional conduct, and patient privacy [31]. Equity-related issues were also a central theme, particularly regarding digital literacy, socioeconomic disparities, and access to technology. While TR has the potential to expand care to underserved populations, it may simultaneously exacerbate disparities: rural populations, older adults, and individuals from lower socioeconomic backgrounds are particularly vulnerable to being excluded from TR services due to technological and geographic barriers, among others. These findings reinforce concerns that TR must be implemented with an equity-driven approach to avoid widening health care access gaps [32].

Additionally, as part of a series of rapid reviews across rehabilitation fields, this study contributes to exploring TR in a multidisciplinary context. Understanding interdisciplinary collaboration in TR is crucial to fully understand the reality of TR and better inform practice, as rehabilitation often involves professionals from diverse fields working together to address complex patient needs. Such collaborative efforts have been shown to improve patient outcomes, enhance service efficiency, and patient satisfaction in rehabilitation [33]. Finally, the use of rapid reviews ensures a timely synthesis of a large body of evidence and current TR practices, enabling researchers to provide actionable results more efficiently.

To ensure the practical impact of this review, our findings will be summarized in targeted formats for different stakeholders, and a structured approach will be used to translate key themes identified in the review into actionable guidance. For example, recommendations for clinicians will be focused on ethical guidelines and best practices for patient engagement, while emphasis will be placed on organizational factors such as implementation strategies, costs, and training requirements for health care administrators. These tailored outputs will support decision-making at various levels, ensuring that TR implementation in neuropsychology and psychology is both effective and equitable.

### Limitations

Despite its strengths, this work has some limitations worth acknowledging. First, the exclusion of gray literature restricts the scope of this rapid review. Information generated outside of traditional scientific peer-reviewed publications, such as government reports, policy briefs, and clinical guidelines, may offer additional insights into the real-world implementation of TR. However, given the substantial amount of scientific literature that is rapidly emerging and the methodological constraints of a rapid review, gray literature was excluded to maintain a rigorous and manageable scope. As a result, some findings may not be fully captured in our synthesis, impacting the applicability of recommendations, particularly in settings where institutional policies and practice-based knowledge play a significant role in shaping TR implementation. Future research could incorporate gray literature to supplement our findings. Moreover, in the context of this rapid review, we are not including telehealth practices in neuropsychology and psychology that are not in the field of rehabilitation (eg, mental health web-based services), although certain learnings from more general telehealth practices could inform TR practices. However, this restriction ensures that our findings are directly applicable to rehabilitation settings, and future studies comparing TR and general telehealth applications could help refine recommendations and make them more adaptable across health care settings. Additionally, the timeframe for included studies was chosen to balance historical perspectives with emerging evidence, particularly given the urgency of generating guidance for TR practices. However, some relevant original research published before 2020 and reviews published after 2020 may have contained relevant information for this review. It is possible that findings regarding ethical and equity considerations outside of these timeframes, such as evolving regulatory frameworks, engagement strategies, or new digital health tools, may not be fully reflected in our findings. This could affect the long-term relevance or our recommendations, reinforcing the need for continuous updates to TR guidelines. Finally, the rapid evolution of digital health technologies and telehealth policies means that TR practices will likely continue to change. As a result, some of the recommendations derived from this review may need adaptation over time.

### Conclusions

In conclusion, the rapid expansion of TR use has presented not only new opportunities but also concerns about quality of care and equitable access to care in the context of neuropsychology and psychology interventions. This study thus aims, through this rapid review, to identify the impact of virtual care in psychology and neuropsychology on equity of access, patients’ and caregivers’ experiences, appropriate use, and the impact of TR on health care. Data emerging from this rapid review will be useful to better understand the issue at stake, namely TR practices in the context of neuropsychology and psychology practices, and along with the next steps of our study, contribute to better TR practices and improved rehabilitation care. Practical, evidence-based recommendations will be developed to guide the appropriate implementation of TR in neuropsychology and psychology. The findings from this study will allow us to cocreate impactful evidence-informed and practice-based recommendations and tools to guide clinical practice and policy and inform both clinical practice and broader health care decision-making. Ultimately, these will support the continued use of TR, inform policy and regulations, and ultimately optimize the health and well-being of people with disabilities.

## Appendix

Multimedia Appendix 1 Full search strategy.

Multimedia Appendix 2 PRISMA (Preferred Reporting Items for Systematic Reviews and Meta-Analyses) checklist.

## Abbreviations

PICOTSS: Population, Intervention, Comparison, Outcome, Timeframe, Study Design, and Setting
PRISMA: Preferred Reporting Items for Systematic Reviews and Meta-Analyses
TR: telerehabilitation
