# Supplementary material for: Paving the Road for More Ethical and Equitable Policies and Practices in Telerehabilitation in Psychology and Neuropsychology: Protocol for a Rapid Review
Source: JMIR Res Protoc. 2025 Apr 22;14:e66639. doi: 10.2196/66639 (PMC12056424; doi:10.2196/66639)
Supplement: Multimedia Appendix 1 [file resprot_v14i1e66639_app1.docx]

Search strategy - Medline (Ovid)

Ovid MEDLINE(R) ALL <1946 to March 27, 2023>

# Searches Results

1 Telerehabilitation/ 927

2 (((digital or web or online or virtual or internet or remote) adj2 rehab*) or telerehab* or tele-rehab* or telept or tele-pt or telespeech or tele-speech or teletherap* or tele-therap* or erehabilitation or e-rehabilitation).tw,kf. 4714

3 1 or 2 4905

4 limit 3 to yr="2020 - 2023" 1891

5 limit 4 to (english or french) 1849

6 Telemedicine/ 36563

7 Remote Consultation/ 5680

8 exp Videoconferencing/ 2718

9 Internet-Based Intervention/ 1087

10 (((digital or web or online or virtual or internet or remote) adj2 (intervention* or consult* or therap*)) or online health* or virtual care or videoconferenc* or video conferenc* or telemedicine or tele-medicine or telehealth* or tele health* or ehealth* or e-health* or teleconsultation* or tele-consultation* or econsultation* or e-consultation* or telecare or tele-care or teleintervention* or tele-intervention* or teletreatment* or tele-treatment* or telepractice* or tele-practice*).tw,kf. 60202

11 or/6-10 78563

12 *Speech-Language Pathology/ or *Audiologists/ or *Language Therapy/ or *Speech Therapy/ 7693

13 (((speech or language or voice) adj2 therap*) or ((speech or language) adj2 patholog*) or audiologist*).ti,kf. 4982

14 (((speech or language or voice) adj2 therap*) or ((speech or language) adj2 patholog*) or audiologist*).ab. /freq=2 4570

15 or/12-14 12198

16 11 and 15 423

17 limit 16 to yr="2020 - 2023" 231

18 limit 17 to (english or french) 227

19 *Occupational Therapy/ or *Occupational Therapists/ or *occupational therapy department, hospital/ 11558

20 (ergotherap* or (occupational adj2 therap*)).ti,kw. 6488

21 (ergotherap* or (occupational adj2 therap*)).ab. /freq=2 6113

22 or/19-21 14768

23 11 and 22 192

24 limit 23 to yr="2020 - 2023" 108

25 limit 24 to (english or french) 108

26 *Physical Therapy Modalities/ or *Physical Therapists/ or *Physical Therapy Specialty/ or *physical therapy department, hospital/ 26774

27 (rehab* or physiotherap* or (physical adj2 therap*)).ti,kw. 112800

28 (rehab* or physiotherap* or (physical adj2 therap*)).ab. /freq=2 84678

29 or/26-28 160805

30 11 and 29 2483

31 limit 30 to yr="2020 - 2023" 1381

32 limit 31 to (english or french) 1346

33 *Neuropsychology/ or *Psychology/ 14287

34 (neuropsycholog* or psycholog*).ti,kf. 143698

35 (neuropsycholog* or psycholog*).ab. /freq=2 121417

36 or/33-35 218040

37 11 and 36 2266

38 limit 37 to yr="2020 - 2023" 1472

39 limit 38 to (english or french) 1453

40 5 or 18 or 25 or 32 or 39 4303

Search strategy - CINAHL (Ebsco)

# Query Results

1 (MH "Telerehabilitation") OR TI ( (((digital or web or online or virtual or internet or remote) N2 rehab*) or telerehab* or tele-rehab* or telept or tele-pt or telespeech or tele-speech or teletherap* or tele-therap* or erehabilitation or e-rehabilitation) ) OR AB ( (((digital or web or online or virtual or internet or remote) N2 rehab*) or telerehab* or tele-rehab* or telept or tele-pt or telespeech or tele-speech or teletherap* or tele-therap* or erehabilitation or e-rehabilitation) ) Limiters - Published Date: 20200101-20231231; Language: English, French 996

2 ( (MH "Telehealth") OR (MH "Telemedicine") OR (MH "Remote Consultation") ) OR (MH "Videoconferencing+") OR (MH "Internet-Based Intervention") 36,326

3 TI ( (((digital or web or online or virtual or internet or remote) N2 (intervention* or consult* or therap*)) or online health* or virtual care or videoconferenc* or video conferenc* or telemedicine or tele-medicine or telehealth* or tele health* or ehealth* or e-health* or teleconsultation* or tele-consultation* or econsultation* or e-consultation* or telecare or tele-care or teleintervention* or tele-intervention* or teletreatment* or tele-treatment* or telepractice* or tele-practice*) ) OR AB ( (((digital or web or online or virtual or internet or remote) N2 (intervention* or consult* or therap*)) or online health* or virtual care or videoconferenc* or video conferenc* or telemedicine or tele-medicine or telehealth* or tele health* or ehealth* or e-health* or teleconsultation* or tele-consultation* or econsultation* or e-consultation* or telecare or tele-care or teleintervention* or tele-intervention* or teletreatment* or tele-treatment* or telepractice* or tele-practice*) ) 39,3

4 S2 OR S3 59,884

5 ( ( (MM "Speech-Language Pathology") OR (MM "Speech-Language Pathologists") OR (MM "Audiology") OR (MM "Audiologists") OR (MM "Speech Therapy") OR (MM "Language Therapy") ) ) OR TI ( (((speech or language or voice) N2 therap*) or ((speech or language) N2 patholog*) or audiologist*) ) OR AB ( (((speech or language or voice) N2 therap*) or ((speech or language) N2 patholog*) or audiologist*) ) 25,942

6 S4 AND S5

Limiters - Published Date: 20200101-20231231; Language: English, French 370

7 ( ( (MM "Occupational Therapy") OR (MM "Occupational Therapists") OR (MM "Occupational Therapy Service") ) ) OR TI ( (ergotherap* or (occupational N2 therap*)) ) OR AB ( (ergotherap* or (occupational N2 therap*)) ) 39,84

8 S4 AND S7

Limiters - Published Date: 20200101-20231231; Language: English, French 286

9 ( (MM "Physical Therapy") OR (MM "Physical Therapists") OR (MM "Physical Therapy Service") ) OR TI ( (rehab* or physiotherap* or (physical N2 therap*)) ) OR AB ( (rehab* or physiotherap* or (physical N2 therap*)) ) 171,165

10 S4 AND S9

Limiters - Published Date: 20200101-20231231; Language: English, French 1,168

11 (MM "Psychology+") OR (MM "Neuropsychology") OR TI ( (neuropsycholog* or psycholog*) ) OR AB ( (neuropsycholog* or psycholog*) ) 175,96

12 S4 AND S11

Limiters - Published Date: 20200101-20231231; Language: English, French 1,298

13 S1 OR S6 OR S8 OR S10 OR S12 3,534
