# Supplementary material for: Paving the Road for More Ethical and Equitable Policies and Practices in Telerehabilitation in Psychology and Neuropsychology: Protocol for a Rapid Review
Source: JMIR Res Protoc. 2025 Apr 22;14:e66639. doi: 10.2196/66639 (PMC12056424; doi:10.2196/66639)
Supplement: Multimedia Appendix 2 [file resprot_v14i1e66639_app2.pdf]

PRISMA-P (Preferred Reporting Items for Systematic review and Meta-Analysis Protocols) 2015 checklist: recommended items to address in a systematic review protocol*

Section and Topic	Item No	Checklist Item
**ADMINISTRATIVE INFORMATION**		
Title:		
Identification	1a	Paving the road for more ethical and equitable policies and practices in telerehabilitation in psychology and neuropsychology: a research protocol for a rapid review
Update	1b	Not applicable
Registration	2	Not applicable
Authors:		
Contact	3a	***Dorothée Morand-Grondin, BSc,*** *Département de psychologie, Université de Montréal, Québec, Canada, dorothee.morand-grondin@umontreal.ca*<br>***Jeanne Berthod, BSc,*** *École de psychologie, Université Laval, Québec, Canada, jeanne.berthod1@psy.ulaval.ca*<br>***Jennifer Sigouin, PhD,*** *Centre de recherche interdisciplinaire en réadaptation du Montréal métropolitain (CRIR), Montreal, Canada, jennifer.sigouin@mail.mcgill.ca*<br>***Simon Beaulieu-Bonneau, PhD****, École de psychologie, Université Laval, Québec, Canada, simon.beaulieu-bonneau@psy.ulaval.ca*<br>***Dahlia Kairy PhD****, Programme de physiothérapie, École de réadaptation, Faculté de Médecine, Université de Montréal, Montreal, Canada, dahlia.kairy@umontreal.ca*<br>Corresponding author: **Dorothée Morand-Grondin**, Département de psychologie, Université de Montréal, Québec, Canada, 90 Ave. Vincent-D'Indy, H2V 2S9, Email: dorothee.morand-grondin@umontreal.ca
Contributions	3b	Dahlia Kairy and Jennifer Sigouin developed the protocol.<br>Dorothée Morand-Grondin and Jeanne Berthod led the rapid review (data screening and extraction).<br>Dorothée Morand-Grondin wrote the first draft and subsequent versions of the manuscript.

		Jeanne Berthod, Jennifer Sigouin, Simon Beaulieu-Bonneau and Dahlia Kairy provided editing and comments for the manuscript.
Amendments	4	Not applicable
Support:		
Sources	5a	This project is funded by the Institut de recherche en santé du Canada, the Association médicale canadienne (CMA) and the Réseau québécois de recherche en adaptation-réadaptation (REPAR).
Sponsor	5b	Not applicable
Role of sponsor or fundor	5c	Funders played no role in developing the protocol and do not have a role in conducting the study.
**INTRODUCTION**		
Rationale	6	In the last years, the use of telerehabilitation has rapidly increased. This delivery care mode has been shown to be as effective as in-person care and has additionally been suggested as a mean to enhance accessibility to healthcare services. Furthermore, TR can improve access to care for remote populations, those with mobility challenges and individuals facing demanding work or caregiving responsibilities. However, complete and consistent guidance is required for clinicians to be able to offer equitable and ethical TR services. TR is a care model that is still in development, and as such, inconsistent and incomplete guidance can potentially affect the quality of care provided, especially for the most vulnerable among our society. Managers and decision-makers are also faced with challenging decisions when considering larger implementation of TR. Additionally, people with disabilities need support and resources to adapt to this new way of receiving rehabilitation services. Therefore, to harness the potential benefits of TR, it is essential to develop and apply robust guidance and tools addressing concerns related to inadequate and inequitable practices. This includes that significant ethical issues need to be addressed for both clinicians and patients, such as proactively identifying and addressing potential inequities adversely affecting certain populations.
Objectives	7	The proposed rapid review is one of a series of rapid reviews which are part of a larger cross-Canada study aimed at guiding policy-making and clinical practice to provide ethically sound and equitable virtual rehabilitation care. To better inform recommendations, a series of rapid reviews, conducted by field of TR, including in

		neuropsychology and psychology are being conducted. Findings from the individual reviews will be combined to inform subsequent steps of the pan-Canadian study (surveys, interviews, focus groups).
**METHODS**		
Eligibility criteria	8	Studies will be included in this review if the meet the following inclusion criteria (1) review article published between 2010 and 2020 as well as original studies published between 2020 and 2023, (2) articles written in French or English, and (3) addressing TR and related terms such as digital health, web, online, virtual, online, internet or remote rehabilitation, with main focus on neuropsychological and/or psychological evaluation and intervention rehabilitation activities conducted with patients with physical disability. These criteria were chosen in order to identify the most up-to-date findings in virtual rehabilitation. Only review studies will be included prior to 2019, whereas reviews and original articles will be included from 2020 to 2023. The latter time frame will allow inclusion of more recent studies, including studies using newer technologies or those taking place during the Covid-19 pandemic.
Information sources	9	Guided by an experienced health-science librarian, an online search strategy will be designed for articles related to virtual care using specific keywords (e.g., telehealth, e-health, digital health, m-health) in three databases (Medline, CINAHL, and EMBASE).
Search strategy	10	Medline (Ovid)<br>Ovid MEDLINE(R) ALL <1946 to March 27, 2023><br>#Searches Results<br>1Telerehabilitation/927<br>2(((digital or web or online or virtual or internet or remote) adj2 rehab*) or telerehab* or tele-rehab* or telept or tele-pt or telespeech or tele-speech or teletherap* or tele-therap* or erehabilitation or e-rehabilitation).tw,kf. 4714<br>31 or 2 4905<br>4limit 3 to yr="2020 - 2023"1891<br>5limit 4 to (english or french)1849<br>6Telemedicine/36563<br>7Remote Consultation/5680<br>8exp Videoconferencing/2718<br>9Internet-Based Intervention/1087

	10(((digital or web or online or virtual or internet or remote) adj2 (intervention* or consult* or therap*)) or online health* or virtual care or videoconferenc* or video conferenc* or telemedicine or tele-medicine or telehealth* or tele health* or ehealth* or e-health* or teleconsultation* or tele-consultation* or econsultation* or e-consultation* or telecare or tele-care or teleintervention* or tele-intervention* or teletreatment* or tele-treatment* or telepractice* or tele-practice*).tw,kf.60202<br>11or/6-10 78563<br>12*Speech-Language Pathology/ or *Audiologists/ or *Language Therapy/ or *Speech Therapy/7693<br>13(((speech or language or voice) adj2 therap*) or ((speech or language) adj2 patholog*) or audiologist*).ti,kf.4982<br>14(((speech or language or voice) adj2 therap*) or ((speech or language) adj2 patholog*) or audiologist*).ab. /freq=24570<br>15or/12-14 12198<br>1611 and 15 423<br>17limit 16 to yr="2020 - 2023"231<br>18limit 17 to (english or french)227<br>19*Occupational Therapy/ or *Occupational Therapists/ or *occupational therapy department, hospital/11558<br>20(ergotherap* or (occupational adj2 therap*)).ti,kw.6488<br>21(ergotherap* or (occupational adj2 therap*)).ab. /freq=26113<br>22or/19-21 14768<br>2311 and 22 192<br>24limit 23 to yr="2020 - 2023"108<br>25limit 24 to (english or french)108<br>26*Physical Therapy Modalities/ or *Physical Therapists/ or *Physical Therapy Specialty/ or *physical therapy department, hospital/26774<br>27(rehab* or physiotherap* or (physical adj2 therap*)).ti,kw.112800<br>28(rehab* or physiotherap* or (physical adj2 therap*)).ab. /freq=284678<br>29or/26-28 160805<br>3011 and 29 2483<br>31limit 30 to yr="2020 - 2023"1381<br>32limit 31 to (english or french)1346

33*Neuropsychology/ or *Psychology/14287
34(neuropsycholog* or psycholog*).ti,kf.143698
35(neuropsycholog* or psycholog*).ab. /freq=2121417
36or/33-35 218040
3711 and 36 2266
38limit 37 to yr="2020 - 2023"1472
39limit 38 to (english or french)1453
405 or 18 or 25 or 32 or 394303

Stratégie - CINAHL (Ebsco)
#Query Results
1(MH "Telerehabilitation") OR TI ((((digital or web or online or virtual or internet or remote) N2 rehab*) or telerehab* or tele-rehab* or telept or tele-pt or telespeech or tele-speech or teletherap* or tele-therap* or erehabilitation or e-rehabilitation)) OR AB ((((digital or web or online or virtual or internet or remote) N2 rehab*) or telerehab* or tele-rehab* or telept or tele-pt or telespeech or tele-speech or teletherap* or tele-therap* or erehabilitation or e-rehabilitation)) Limiters - Published Date: 20200101-20231231; Language: English, French996
2((MH "Telehealth") OR (MH "Telemedicine") OR (MH "Remote Consultation")) OR (MH "Videoconferencing+") OR (MH "Internet-Based Intervention")36,326
3TI ((((digital or web or online or virtual or internet or remote) N2 (intervention* or consult* or therap*)) or online health* or virtual care or videoconferenc* or video conferenc* or telemedicine or tele-medicine or telehealth* or tele health* or ehealth* or e-health* or teleconsultation* or tele-consultation* or econsultation* or e-consultation* or telecare or tele-care or teleintervention* or tele-intervention* or teletreatment* or tele-treatment* or telepractice* or tele-practice*)) OR AB ((((digital or web or online or virtual or internet or remote) N2 (intervention* or consult* or therap*)) or online health* or virtual care or videoconferenc* or video conferenc* or telemedicine or tele-medicine or telehealth* or tele health* or ehealth* or e-health* or teleconsultation* or tele-consultation* or econsultation* or e-consultation* or telecare or tele-care or teleintervention* or tele-intervention* or teletreatment* or tele-treatment* or telepractice* or tele-practice*))39,3
4S2 OR S3 59,884

		5(((MM "Speech-Language Pathology") OR (MM "Speech-Language Pathologists") OR (MM "Audiology") OR (MM "Audiologists") OR (MM "Speech Therapy") OR (MM "Language Therapy"))) OR TI ((((speech or language or voice) N2 therap*) or ((speech or language) N2 patholog*) or audiologist*)) OR AB ((((speech or language or voice) N2 therap*) or ((speech or language) N2 patholog*) or audiologist*)) 25,942<br>6S4 AND S5<br>Limiters - Published Date: 20200101-20231231; Language: English, French370<br>7(((MM "Occupational Therapy") OR (MM "Occupational Therapists") OR (MM "Occupational Therapy Service"))) OR TI ((ergotherap* or (occupational N2 therap*))) OR AB ((ergotherap* or (occupational N2 therap*)))39,84<br>8S4 AND S7<br>Limiters - Published Date: 20200101-20231231; Language: English, French286<br>9((MM "Physical Therapy") OR (MM "Physical Therapists") OR (MM "Physical Therapy Service")) OR TI ((rehab* or physiotherap* or (physical N2 therap*))) OR AB ((rehab* or physiotherap* or (physical N2 therap*)))171,165<br>10S4 AND S9<br>Limiters - Published Date: 20200101-20231231; Language: English, French1,168<br>11(MM "Psychology+") OR (MM "Neuropsychology") OR TI ((neuropsycholog* or psycholog*)) OR AB ((neuropsycholog* or psycholog*))175,96<br>12S4 AND S11<br>Limiters - Published Date: 20200101-20231231; Language: English, French1,298<br>13S1 OR S6 OR S8 OR S10 OR S123,534
Study records:		
Data management	11a	The data collection and extraction will take place using the screening and extraction software Covidence.
Selection Process	11b	Studies will be screened, chosen and appraised independently by 2 reviewers and discrepancies resolved through discussion. More precisely, after identifying reviews and articles using the keywords, the data collection will follow three main steps. First, the reviews and articles will be screened based on their title and abstract. Second, the reviews and articles included in the first step will be examined by the reviewers based on the full text. Third, the data will be extracted from the included reviews and articles in data extraction tables and analysed with team members for emerging and overarching themes. The Equity-Based Framework for

		Implementation Research, the Quadripartite Ethical Tool and the Consolidated Framework for Implementation Research will be used to guide the data collection and analysis.
Data Collection Process	11c	Data will be extracted using a customized data extraction spreadsheet developed by one of the authors (JS) based on the study frameworks and adapted to the needs of psychological and neuropsychological fields by two of the authors (JB and SBB). An independent verification of the data extraction was performed by a second author on approximately 5% of the articles.
Data items	12	Characteristics of interest include (1) the field of practice, (2) the clinical features (e.g., patient population, technology used, outcome measures and tools used, frequency of intervention), (3) study objectives and findings, (4) the limitations (e.g., ethical limitations), (5) the quality assessment and risk of bias and (6) equity considerations.
Outcomes and prioritization	13	Studies included will be examined for possible bias for the relevant outcomes as suggested by the Cochrane Rapid reviews Methods Group. For example, risk of bias ratings will be limited to the primary outcomes.
Risk of bias in individual studies	14	We address two central questions on quality assessment, adapted from the systematic review appraisal guidelines by the University of Oxford's Centre for Evidence-Based Medicine: a) Were all relevant studies included? and b) Were the criteria used to select articles for inclusion and exclusion appropriate, or at risk of introducing a bias? Additionally, we recorded whether each review employed a quality assessment method for evaluating the articles incorporated in their reviews.
Data synthesis	15a	Not applicable
	15b	Not applicable
	15c	Not applicable
	15d	Data will be synthesized according to the most common ethical and equity issues that were either raised by the authors or unaddressed by majority of authors in order to identify gaps in the literature.
Meta-bias(es)	16	Not applicable
Confidence in cumulative evidence	17	Not applicable

https://static1.squarespace.com/static/65b880e13b6ca75573dfe217/t/65b9e4128a67f31f64b09aeb/1706681363097/PRISMA-P-checklist.pdf
